# Technoeconomic data adopted for the development of a long-term electricity supply model for the Hashmite Kingdome of Jordan

**DOI:** 10.1016/j.dib.2020.105391

**Published:** 2020-03-07

**Authors:** Osama Saadeh, Zakariya Dalala, Taco Niet, Eunice Pereira Ramos, Mark Howells

**Affiliations:** a German Jordanian University, Jordan; b Simon Fraser University, Canada; c KTH Royal Institute of Technology, Sweden; d Loughborough University, UK

**Keywords:** Energy policy, Renewable energy, Cost-optimisation, Jordan scenarios

## Abstract

Electrical generation in Jordan currently relies on imported fossil fuels. In the past, most imported fossil fuels were subsidised by neighbouring countries through grants and aid. This has led to a regulated market, with subsidised low-cost electrical energy consumers, and the government being the sole buyer and seller of electricity. With the ageing of the national electrical infrastructure, political instability in the region, and lack of funds for direct investment, other options needed to be pursued. Long term Power Purchase Agreements (PPA) were granted to Independent Power Producers (IPP) to encourage investment in capacity and infrastructure. In addition, long-term fuel contracts were signed to secure steady flow of primary fuel sources. Over the past few years, renewable energy penetration has increased rapidly, but without proper planning or taking into consideration long term PPA and fuel contracts. Data in regard to the current infrastructure, renewable energy technology, signed energy commitments and system operation assumptions are described in this article, which may be used for modelling and analysis. The Data were collected from annual reports from the different energy related entities in Jordan.

Specifications table

**Table:** 

Subject	Renewable Energy and Sustainability
Specific subject area	Renewable energy assessment and primary fuel sources.
Type of data	Tables and Graphs
How data were acquired	Databases and reports of international and domestic organisations
Data format	Raw and analysed
Parameters for data collection	Data collected based on inputs required to understand the impact of renewable energy penetration on the reliability of electric delivery in Jordan.
Description of data collection	Data were collected from annual reports from the different energy related entities in Jordan.
Data source location	Country: Hashemite Kingdome of Jordan
Data accessibility	With the article

## Value of the data

• The data provided are required to understand the impact of renewable energy penetration to the reliability of electric delivery in Jordan.
• Researchers working on modelling to assist systems planners can use the provided data to better plan future expansion of conventional generation as well as quantifying renewable energy penetration levels.
• The data can be used as a standard model input, which will help define what other data sets may be required for more detailed modelling.

## 1 Data description

The data provided in this paper are used for the development and analysis of an energy model for Jordan. The datasets presented were collected from different energy related entities in Jordan. It includes electrical energy consumption, power demand, generated energy, fuel sources, generation technologies, installed capacity and future projects.

The datasets are provided with this article in the form of tables and a figure, which are described in Table 1.

**Table 1:** Description of items and datasets provided with article.

Item	Title	Description of content
1.	Table 2	A table that list the percentage of electrical energy consumption per sector in Jordan.
2.	Table 3	A table that lists projected peak power demand and generated energy from 2020 to 2040.
3.	Table 4	A table listing installed capacity from all different technologies connected to the Jordanian electric network from 2016 to 2019.
4.	Table 5	A table that list projected fuel prices from 2020 to 2050.
5.	Fig. 1.	A graph showing generation cost comparison for CCGT, solar PV and wind from 2015 to 2045.
6.	Table 6	A table that lists plant cost and performance parameters for future investment for all generation technologies.
7.	Table 7	A table showing all current and contracted PV plants from 2020 to 2023. Table includes capacity, predicted energy generation, initial investment and operation cost.
8.	Table 8	A table showing all current and contracted wind power plants from 2020 to 2023. Table includes capacity, predicted energy generation, initial investment and operation cost.

### 1.1 Electricity supply and generation data

Jordan is a small country that primarily relies on imported fuel for all electric energy generation, the government serves as a single buyer of primary fuel sources [1]. The current electrical generation installed capacity in Jordan is 4.3 GW [2]. The consumption of electricity based on sector for 2018 is shown in Table 2, and projected peak power demand and generated energy up to year 2040 are shown in Table 3.

**Table 2:** Electrical energy consumption per sector [4].

Sector	Residential	Street Lights	Agricultural and Water Pump	Commercial and Hotels	Industrial
Percent Consumption	45.12	2.32	15.4	15.08	22.08

**Table 3:** Projected peak power demand and generated energy [4].

Year	Peak Power Demand		Generated Electrical Energy	
	MW	% growth	GWh	% growth
2020	3146	2.9	20,744	3.0
2022	3341	3.1	22,063	3.2
2025	3645	2.9	24,250	3.2
2030	4186	2.8	28,230	3.1
2040	5528	2.8	38,261	3.1

Jordan's peak load in 2016 was 3500 MW, with an installed capacity of 3800 MW from traditional generation, excluding renewable energy penetration. Transmission level Renewable energy installed capacity was approximately 407 MW [1,^2^,^4^,5]. In addition, 200 MW of distribution level net-metering PV systems are also operational. Table 4 details installed capacity from all generation technologies.

**Table 4:** Installed capacity in the Jordanian electric network.

	2016	2017	2018	2019
CCGT Installed Capacity (MW)	3800	4200	4200	4200
PV Capacity (MW)	209.8	312.8	648.8	1028.8
Wind Capacity (MW)	197	197	372.2	616.2
Total Renewable Capacity (MW)	407	510	1061	1645
PV share to system Capacity (%)	5.00	6.60	12.30	17.60
Wind share to system Capacity (%)	4.70	4.20	7.10	10.50
Total Renewable share to system Capacity (%)	10	11	20	28

### 1.2 Imported fuel

Data from the Jordanian Energy Information System and the U.S. Energy Information Administration are used to compile projected fuel prices as shown in Table 5 [1,6]. Only data for fuel sources used in Jordan are considered.

**Table 5:** Projected fuel prices [6].

		2020	2025	2030	2035	2040	2045	2050
Oil	USD/barrel	77.1	97.2	123.5	148.4	173.9	199.8	225.7
	USD/MMBtu	13.3	16.8	21.3	25.6	30	34.4	38.9
Natural Gas Contract Price	USD/MMBtu	6.3	6.3	6.3	6.3	6.3	6.3	–
Natural Gas at Henry Hub	USD/MMBtu	3.2	4.2	5	6	7	8.3	10.2

### 1.3 Power plant costs and performance data

**Fig. 1:**
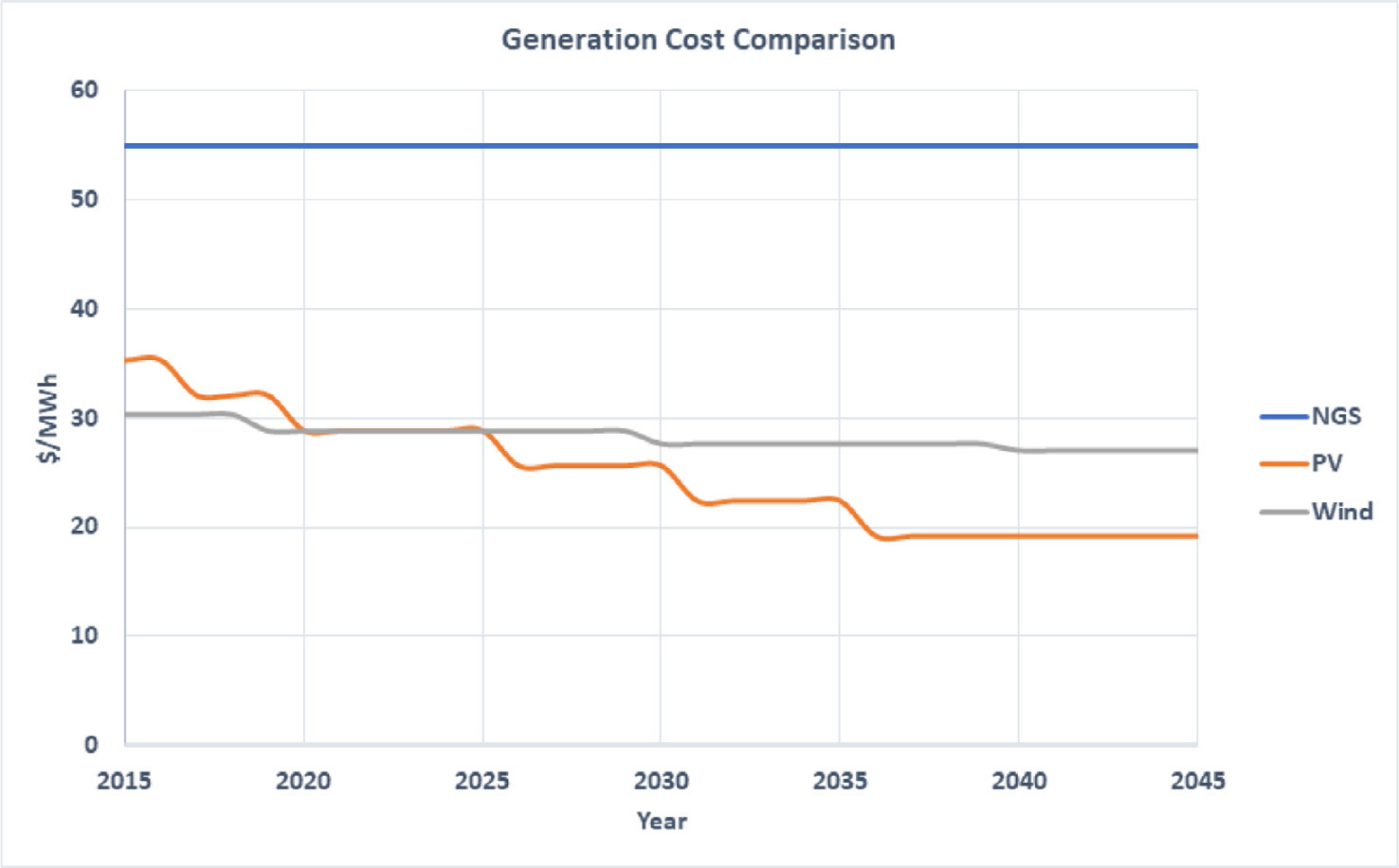
Generation cost comparison for CCGT, solar PV and wind.

The initial investment costs for solar PV projects are 1000 USD/kWp and 2020 USD/kW for wind projects [2,^4^,5]. Fig. 1 shows the generation cost of the different technologies up to year 2045. Table 6 lists plant cost and performance parameters for future investment for all generation technologies. Table 7 shows all current and contracted PV plants from 2020 to 2023. The table includes capacity, predicted energy generation, initial investment and operation cost. Table 8 showing all current and contracted wind power plants from 2020 to 2023. The table includes capacity, predicted energy generation, initial investment and operation cost [7].

**Table 6:** Plant cost and performance parameters for future investment.

Technology Type	Input Fuel	Efficiency	Variable Operation and Maintenance cost (USD/MWh)	Fixed operation and maintenance cost (USD/kW)	2019 investment cost (USD/kW)	Capacity factor	Construction time (years)	Plant Life (years)
Combined Cycle Gas turbines	Natural Gas	47.5		37.2	930	86	3	30
Wind			8.4		1920	32	1	20
Solar PV utility			8.4		900	28	1	25
Solar PV rooftop			6		1000	28	1	25

**Table 7:** Future PV planst specifications.

Year of Operation	2020	2021	2022	2023
Additional installed Capacity (MW)	306	200	40	30
Predicated New Generated Energy (GWh)	477.4	312	62.4	46.8
Capital Cost (USD/kW)	900	900	900	900
Variable Operation and Maintenance cost (USD/MWh)	8.4	8.4	8.4	8.4

**Table 8:** Future wind plants specifications.

Year of Operation	2020	2021	2022	2023
Additional installed Capacity (MW)	551	200	40	80
Predicated New Generated Energy (GWh)	1836.5	666.6	133.3	266.6
Capital Cost (USD/kW)	1920	1920	1920	1920
Variable Operation and Maintenance cost (USD/MWh)	8.4	8.4	8.4	8.4

## 2 Experimental design, materials and methods

Data were collected from annual reports provided by the Energy and Mineral Regulation Commission, the National Electric Power Company (NEPCO), in addition to independent power producers (IPPs) and recent commissioned PV projects in Jordan.

### 2.1 Electricity supply and generation data

NEPCO is the sole buyer of bulk electricity from the IPPs. NEPCO then sells the bulk power to three geographically distributed, distribution companies, which provide electricity to consumers. Data in Table 2 were collected from NEPCO's annual report as it includes data from all the distribution companies. Data in Table 3 were collected form the Energy and Mineral Regulation Commission, which tabulated information from all IPPs. Information in Table 4 was collected from the individual IPP annual reports for the traditional generation capacity, as each individual IPP provides the installed capacity connected to the Jordanian Electric Network. While renewable energy data were collected from the International Renewable Energy Agency, which provides data on installed capacity and contracted projects and from the Energy and Mineral Regulation Commission.

### 2.2 Fuel data

The Jordanian government serves as a single buyer of primary fuel sources, through the government owned Transmission Company NEPCO. NEPCO sells fuel to IPPs at a fixed rate regardless of market prices, in exchange for a fixed generation cost. As the political climate in the region caused a turmoil in gas supplies from Egypt and Arabian Gulf countries, NEPCO signed a 15 year contract to import gas from the Leviathan natural gas field. The contract is to import 3 billion cubic meters for a total contract price of 10 billion USD over the contract period. Projected fuel cost in Table 5 was obtained from the Jordanian Energy Information System and the U.S. Energy Information Administration.

### 2.3 Power plant costs and performance data

As renewable energy prices dramatically decreased starting in 2010, deployment of renewable energy projects through PPAs in Jordan increased drastically [3]. However, Data required for modelling and analysis includes initial investment, in addition to operation and maintenance cost. The Data in Tables 6–8 were compiled from data available in NEPCO's annual report, the International Renewable Energy Agency roadmap, in addition to recently signed contracts in Jordan, which provided actual recent cost [7].

## Appendix Supplementary materials

Image, application 1

Image, application 2
